# Tinnitus in normally hearing patients: clinical aspects and repercussions

**DOI:** 10.1016/S1808-8694(15)31194-0

**Published:** 2015-10-20

**Authors:** Tanit Ganz Sanchez, Ítalo Roberto Torres de Medeiros, Cristiane Passos Dias Levy, Jeanne da Rosa Oiticica Ramalho, Ricardo Ferreira Bento

**Affiliations:** 1 Full Professor in Otorhinolaryngology, FMUSP, Associate Professor, Discipline of Otorhinolaryngology, FMUSP.; 2 Ph.D. in Medicine, Medical School, University of Sao Paulo, Ph.D. in Medicine, Discipline of Otorhinolaryngology, FMUSP.; 3 Post-graduate studies under course, Intern physician, Group of Tinnitus Research, Hospital das Clínicas da FMUSP.; 4 Ph.D. studies in Otorhinolaryngology under course, Medical School, USP.; 5 Full Professor in Otorhinolaryngology, FMUSP, Associate Professor, Discipline of Otorhinolaryngology, FMUSP. Study carried out in the Division of Clinical Otorhinolaryngology, Hospital das Clínicas da FMUSP.

**Keywords:** tinnitus, normal hearing, quality of life

## Abstract

Patients with tinnitus and normal hearing constitute an important group, given that findings do not suffer influence of the hearing loss. However, this group is rarely studied, so we do not know whether its clinical characteristics and interference in daily life are the same of those of the patients with tinnitus and hearing loss. **Aim**: To compare tinnitus characteristics and interference in daily life among patients with and without hearing loss. **Study design:** historic cohort. **Material and Method**: Among 744 tinnitus patients seen at a Tinnitus Clinic, 55 with normal audiometry were retrospectively evaluated. The control group consisted of 198 patients with tinnitus and hearing loss, following the same protocol. We analyzed the patients’ data as well as the tinnitus characteristics and interference in daily life. **Results**: The mean age of the studied group (43.1 ± 13.4 years) was significantly lower than that of the control group (49.9 ± 14.5 years). In both groups, tinnitus was predominant in women, bilateral, single tone and constant, but there were no differences between both groups. The interference in concentration and emotional status (25.5% and 36.4%) was significantly lower in the studied group than that of the control group (46% and 61.6%), but it did not happen in regard to interference over sleep and social life. **Conclusions**: Patients with tinnitus and normal hearing showed similar characteristics when compared to those with hearing loss. However, the age of the patients and the interference over concentration and emotional status were significantly lower in this group.

## INTRODUCTION

Tinnitus is one of the most intriguing symptoms in Otorhinolaryngology. On the one hand, it presents a large prevalence (15% in the general population and 33% in the elderly)^1^ and causes considerable morbidity, which may interfere in sleep, concentration, emotional balance and social life of subjects 2, 3. On the other hand, complexity of pathophysiology and subjectivity reduces the interest of Otorhinolaryngologists in this symptom.

The association between tinnitus and hearing loss has been well described. According to different reports, 85 to 96% of the patients with tinnitus present some level of hearing loss 4, 5, 6, 7, 8 and only 8 to 10% present normal audiometry ^9^. In this last group, the isolated presence of tinnitus suggests that it may be a primary symptom of diseases that are only diagnosed after the onset of hearing loss. The origin of tinnitus in these cases is still more obscure than in those with concomitant hearing loss. Thus, despite rare, these patients form a significant sample owing to the characteristics attributed to tinnitus, and not to hearing loss that follows the other cases.

The limited literature on tinnitus cases with normal audiometry is restricted to the study of otoacoustic emissions 10, 11, 12, 13, 14, ABR ^15^, auditory processing 16, 17, 18, high frequency audiometry ^19^ and zinc deficiency ^20^. We did not find studies that approached clinical characteristics and repercussions of tinnitus in this minority group of patients or any study that compared them to a group of subjects with hearing loss.

The patients seen in the tinnitus Research group, Ambulatory of Otorhinolaryngology, Hospital das Clínicas, FMUSP are routinely submitted to a medical and audiological protocol that allows assessment of clinical characteristics of tinnitus and correlated symptoms, as well as the repercussions of patients’ lives, facilitating the creation of the main diagnostic suspicions and the initial direction to be followed by each case.

In a previous study, we dedicated to investigating clinical and epidemiological characteristics of the 150 first patients seen in the group ^21^. However, in this sample we did not reveal the audiometric results in data interpretation. In past years, we have seen more patients with normal audiometry and we wondered whether this specific group would have clinical characteristics and interference in daily live that could distinguish them from the other subjects with tinnitus. Our hypothesis is that these patients present similar clinical characteristics (duration of disease, location, type, frequency of onset), but interferences in daily life activities (sleep, concentration, emotional status and social activities) are less significant than in those with tinnitus and hearing loss.

By emphasizing the importance of better knowing the group of subjects with tinnitus and normal audiometry, the purposes of the present study are: 1) General - to describe the sample and to compare the proportions of clinical characteristics and interferences in daily lives of patients with tinnitus with and without associated hearing loss, and 2) Specific - to check whether there are differences in the groups concerning clinical characteristics and daily life interferences.

## MATERIAL AND METHOD

### Sample

To the purpose of this cross-section observational study, we retrospectively reviewed the medical charts of patients in the tinnitus Research Group, Ambulatory of Otorhinolaryngology, Medical School, USP, seen according to the same medical and audiological protocol. Patients were divided into 2 groups of the same way:

1. Study group: we included all patients that presented tinnitus in the presence of normal pure tone thresholds in all frequencies (≤ 25 dB HL in 250 to 8000 Hz), seen in the period between 1994 and 2003. We excluded only patients with pulsatile tinnitus.
2. Control Group: we excluded all patients with tinnitus and any level of hearing loss, seen in the tinnitus group between 1994 and 1999, which had already been included in the database and who were used as sample in previous publications 22, 23. We excluded patients with pulsatile tinnitus.

### Studied Variables

We analyzed the following variables based on the routine medical and audiological protocol:

A. of patients:
  A1. age
  A2. sex
B. of clinical characteristics of tinnitus:
  B1. tinnitus duration, randomly classified as: recent (< 5 years), mean duration (between 5 and 10 years) and old (> 10 years)
  B2. location, classified as unilateral or bilateral
  B3. type, classified as single or multiple
  B4. frequency of perception, classified as constant or intermittent
C. repercussion of tinnitus in the life of the patient:
  C1. grade of disturbance according to the analogical-visual scale, classified as mild (0 to 3), moderate (4 to 7) and severe (8 to 10)
  C2. interference in sleep, classified as present or absent C3. interference in concentration, classified as present or absent
  C4. interference in emotional status, classified as present or absent
  C5. interference in social life, classified as present or absent

### Calculation of Sample size and statistical analysis

The calculation of the sample size to compare both proportions (nominal data) was performed by adopting a proportion of success (present event) for the control group of 0.5 (proportion that provided maximum variance), considering that there was no literature data nor pilot study that provided this information. Based on the formulated hypothesis, we expected that the proportion of success for the studied group would be smaller than for the control group, considering that the first one would have less interference of tinnitus in daily life. Considering reasonable to detect the difference of at least 30% in interference of tinnitus in the studied group, with a power test of 80% and level of significance of 5%, we would require 40 patients in each group.

Since groups were not matched, nominal data (gender, location, type, frequency, interference in sleep, concentration, emotional status, social activities) and ordinal data (tinnitus duration and disturbance grade) were statistically analyzed by the tables of contingence 2x2 and 3x2 and submitted to chi-square test. Continuous data (age) were submitted to Kolmogorov-Smirnov test to check normal distribution, and later to t test for non-matched samples.

## RESULTS

The study group was comprised by 55 patients who had tinnitus and normal audiometry among the 744 patients seen between 1994 and 2003, corresponding to a percentage of 7.4% of the total of patients. The control group, in turn, comprised 198 patients who had tinnitus associated with hearing loss seen between 1994-1999.

**A.** Concerning patients’ variables, we observed that:
  **A1.** mean age was 43.1 ± 13.4 years in the studied group [IC 95%= 39.5 to 46.7 years] and 49.9 ± 14.5 years in the control group [IC 95%= 47.8 to 51.8 years], presenting normal distribution by Kolmogorov-Smirnov test. There was significant difference between means of both group (p=0.0021), with mean difference of 6.8 years and confidence interval of 95% for the difference = 2.5 to 11.
  **A2.** the proportion of women was greater in both groups, divided as 67.3% in the studied group and 55.6% in the control group. However, there was no significant difference between the groups (p=0.1192).
**B.** As to tinnitus variables:
  **B1.** duration: tinnitus that started less than 5 years before were the most frequent in both groups (56.4% in the studied group and 65.2% in the control group), but there was no statistically significant difference between them (p=0.5371).
  **B2.** location: The distribution of location of tinnitus in the control group was bilateral in 50.9% of the cases, unilateral in 41.8% and on the head in 7.3%. In the control group, the distribution was 42.4%, 52.5% and 5.1%, respectively. There was no statistically significant difference between the groups (p=0.3557).
  **B3.** type: Single tinnitus was more frequent than multiple tinnitus (72.7% in the studied group and 60.1% in the control group), but there was no statistically significant difference between the groups (p=0.1023).
  **B4.** frequency of perception: Constant tinnitus was more frequent than intermittent one (69.1% in the studied group and 73.7% in the control group), but there was no statistically significant difference between the groups (p=0.4937).
**C.** A to variables of the repercussions of tinnitus in the patients’ life:
  **C1.** The level of disturbance of the tinnitus reported by the patient was considered severe in 47.3% of the cases in the studied group and in 45.5% of the control group, without any statistically significant difference (p=0.7853).
  **C2.** The interference of tinnitus in sleep was reported by 58.2% of the patients in the studied group and in 47.5% of the control group, without any statistically significant difference (p=0.1912).
  **C3.** The interference of tinnitus in the concentration was reported in 25.5% of the patients in the studied group and in 46% of the control group, with statistically significant difference between the groups (p=0.0049). The relative risk of this variable was 0.4715, with confidence interval of 95% =0.2714 to 0.8193.
  **C4.** The interference of tinnitus in the emotional status was reported in 36.4% of the patients in the studied group and in 61.6% of the control group, with statistically significant difference between the groups (p=0.005). The relative risk of this variable was 0.4346, with confidence interval of 95%=0.2665 to 0.7089.
  **C5.** The interference of tinnitus in the social activities was reported in 14.5% of the patients in the studied group and in 14.1% in the control group, without any statistically significant difference between the groups (p=0.9722).

## DISCUSSION

Tinnitus is a symptom that may be caused by different otological, metabolic, neurological, cardiovascular, pharmacological, dental and psychological affections, which, in turn, may be present concomitantly in the same subject 2, 3. Despite recent advances in the specific literature, the pathophysiology is still not completely elucidated, which hinders the advancement of its treatment.

Even though tinnitus is a frequently associated symptom with hearing loss detected by pure tone audiometry, it does not happen all the time. In our tinnitus research group, which works as a reference center, the prevalence of tinnitus with normal audiometry was 7.4% out of a total of 744 patients. This percentage is compatible with data by Barnea et al.^9^, confirming that the association with hearing loss is much more frequent.

The interpretation of tinnitus in patients with normal audiometry is quite interesting. The simple experiment by Heller and Bergman, in 1953^24^, demonstrated that 94% of 80 healthy normal hearing subjects perceived tinnitus while in quiet in an anechoic chamber. Thus, it is possible that tinnitus does not always represent that something wrong is going on with the auditory pathways. In these patients specifically, the sensation of tinnitus could have represented the perception of spontaneous activity of the auditory pathway, which was facilitated by the silence of the booth. Conversely, we have seen patients whose tinnitus is the first symptom of otological diseases that are more easily diagnosed after the installation of hearing loss (otospongiosis, sound pressure level induced hearing loss, ototoxicity, vestibular schwannoma, etc.). For this reason, even if tinnitus can have the meaning shown by Heller and Bergman, we believe that hearing in these patients should be periodically monitored, in search for early signs of abnormalities.

In our study, mean age of the studied group was statistically younger than the control group. Even though the etiological definition had not been object of study, it would be logical to assume that presbycusis is one of the most frequent causes of tinnitus and hearing loss, which would have contributed to increase the age range of the control group.

In both groups, there was predominance of female patients, which is in agreement with the findings by Mckee and Stephens^10^, who found 61% of women among subjects with normal audiometry.

Bilateral presentation and onset of tinnitus were similar in both groups, but there was predominance of single and constant tinnitus, which confirmed our clinical impression. We did not find any other reference about these characteristics in patients with normal audiometry. The similarity observed between the control and the studied groups suggests that both are homogenous concerning the clinical characteristics of tinnitus, that is, the presence or absence of hearing loss did not influence these characteristics.

The presence of tinnitus frequently becomes a factor that has negative influence in the lives of subjects, hindering their sleep, concentration on daily and professional activities, as well as their social life. In many occasions it affects emotional balance of the patients, triggering or worsening anxiety and depression states 25, 26. Disturbance caused by tinnitus and repercussions in patients’ lives are certainly the determining factor for their seeking for hospitals and clinics, which justifies the high grade they scored in the analogical-visual scales with higher frequency of moderate and severe tinnitus ^21^. However, our service belongs to a tertiary hospital, so we should not extrapolate these data to the general population.

The psychological aspect of tinnitus is well known and has been properly discussed by many authors 10, 21, 25, 27. In our study, the interference of tinnitus in the concentration and emotional status was significantly smaller than in patients with normal audiometry. Barnea et al.^9^ compared subjects with tinnitus with and without hearing loss and also had similar results. Therefore, it is quite reasonable to assume that the presence of hearing loss increases the risk of tinnitus causing interference in concentration and emotional balance or that it works as a co-factor if this interference, or in other words, the disturbance grade given to tinnitus may be contaminated by the disturbance caused by associated hearing loss.

Our study to clarify the etiopathophysiology and progression of groups of patients with normal hearing with tinnitus tends to support its characterization, allowing a more coherent and precise intervention in the clinical approach of these subjects.

## CONCLUSION

Patients with tinnitus and normal audiometry represent a rare group. Clinical characteristics of tinnitus (duration of disease, location, type, frequency of onset) in these patients are similar to those in subjects with tinnitus and hearing loss. However, the interference caused by the concentration and emotional balance was significantly lower, which did not occur in relation to sleep and social activity interference.
